# Supplementary material for: Plasmodium falciparum Niemann-Pick Type C1-Related protein relies on its physicochemical properties for membrane contact site localization required for cholesterol homeostasis
Source: bioRxiv. 2026 Mar 26:2026.03.25.714277. Preprint. [Version 1] doi: 10.64898/2026.03.25.714277 (PMC13042079; doi:10.64898/2026.03.25.714277)
Supplement: Supplement 2 [file NIHPP2026.03.25.714277v1-supplement-2.pdf]

bioRxiv preprint doi: https://doi.org/10.64898/2026.03.25.714277; this version posted March 26, 2026. The copyright holder for this preprint (which was not certified by peer review) is the author/funder, who has granted bioRxiv a license to display the preprint in perpetuity. It is made available under aCC-BY 4.0 International license.

Supplementary Information

Plasmodium falciparum Niemann-Pick Type C1-Related protein relies on its physicochemical properties for membrane contact site localization required for cholesterol homeostasis

Ray et al.

bioRxiv preprint doi: https://doi.org/10.64898/2026.03.25.714277; this version posted March 26, 2026. The copyright holder for this preprint (which was not certified by peer review) is the author/funder, who has granted bioRxiv a license to display the preprint in perpetuity. It is made available under aCC-BY 4.0 International license.

Supplementary Table 1

Primer name	Primer sequence (5’ → 3’)
P1	GAATAAACACGATTTTTTCTCGAGATGTTCGTAAAAAATTTTATACATAAATTAAAAG
P2	GGTTATTCTCATCCAACACTATATTACTAGTACTTTGTTCAGGTGGTAACTC
P3	CTGAGTTACCACCTGAACAAAGTACTAGTAATATAGTGTTGGATGAGAATAACC
P4	CACtcctccacttccgctagcATGTAGTGGGCCAAAACTG
P5	CCCACATTTCGAATAAACACGATTTTTTCTCGAGatgacaagaagatatttaaag
P6	GTTTTATATAATTCTTTTGCCACATGACTAGTacacaatacattattaataac
P7	caagttattaataatgtattgtgtACTAGTCATGTGGCAAAAGAATTATATAAAAC
P8	CACtcctccacttccgctagcTTCCTTTTTCTTTTTTAACATTTC
P9	CTCacctccactacctccaccacttccCGCCAAAGATAAAACACTC
P10	CTTTGGCGggaagtggtggaggtagtggaggtGAGGAAGATGAAGAAAAACAG
P11	CTCATTATTATTATTGTTATCATTCTCATTATTATTATTATTCGCGCTAGTTTC
P12	GAAACTAGCGCGAATAATAATAATAATGAGAATGATAACAATAATAATAATGAGGATG
P17	CATTATTTTCCTTCCCtccacttccacacaatacattattaataacttg
P18	caagttattaataatgtattgtgtggaagtggaGGGAAGGAAAATAATGATG
P19	CACtcctccacttccgctagcTTCATTATCTTCATCATC
P34	CCTTCCCacctccactacctccaccacttccACTTTGTTCAGGTGGTAACTC
P35	CTGAACAAAGTggaagtggtggaggtagtggaggtGGGAAGGAAAATAATGATGATACG
P36	CACTATATTacctccactacctccaccacttccTTCATTATCTTCATCATCATCATCATC
P37	GATAATGAAggaagtggtggaggtagtggaggtAATATAGTGTTGGATGAGAATAACC

bioRxiv preprint doi: https://doi.org/10.64898/2026.03.25.714277; this version posted March 26, 2026. The copyright holder for this preprint (which was not certified by peer review) is the author/funder, who has granted bioRxiv a license to display the preprint in perpetuity. It is made available under aCC-BY 4.0 International license.

P44	GTTTAATACGTTGTggaagtggaggaccatggGGGAAGGAAAATAATGATGATACG
P45	GGAGTCGTATCTTCTTCACTtcctccacttccCTTGTACAGCTCGTCCATG
P46	CATGGACGAGCTGTACAAGggaagtggaggaAGTGAAGAAGATACGACTCC
P47	cgtcgtccttgtagtccctaggTAGGAATTCTATAAATAATAAAAGTAAAATTACTAAG
P48	CGTTGTggaagtggaggaccatggCATGTGGCAAAAGAATTATATAAAACATTAATTC
P49	GACACtcctccacttccgctagcTTCCTTTTTCTTTTTTAACATTTCATAAATTTTTC
P59	CATTTCGAATAAACACGATTTTTTCTCGAGATGAAAATACCGTTTTTTATTTTAC
P60	CCTTCCCccatggtcctccacttccACAACGTATTAAACATAATAAAAATTG
P66	CTTAGACACtcctccacttccgctagcACAACGTATTAAACATAATAAAAATTGTAAT
GFP-1	CTTAGTTCTTTTGGCCCACTACATGACGTCATGAGTAAAGGAGAAGAACTTTTCA C
GFP-2	TGCATAATCAGGTACGTCATAAGGATAGCCGTCTTATTTGTACAGTTCATCCATGC CATG
StAR-1	CGAATAAACACGATTTTTTCTCGAGATGAGTTTAAAAAGAAGAAAATTTATTTTGTTGTCTT TTG
StAR-2	CTTAGACACTCCTCCACTTCCGCTAGCGTCCTTATTAAAAAATATACCAAATATTTTTTTAAA AAAGTTAACG

Supplementary Table 2

H1	PWHVAKELYKTLIQRSEENLKSGKINGSLFDYSDLDENGKSVLSLAAS
H2	PWEEDEEKQKSLREKLYRKIYEMLKKKKEAS
$ATG3_{AH}$-linker-$ATG3_{AH}$	PWMQNVINTVKGKALEVAEYLTGKENNDDTLIEGDNKNDEETSANNNNNEDDNNEDD NNEDDNNEDDNNENDNNNNNEDDNNNDDDDDEDNEMQNVINTVKGKALEVAEYLT AS

bioRxiv preprint doi: https://doi.org/10.64898/2026.03.25.714277; this version posted March 26, 2026. The copyright holder for this preprint (which was not certified by peer review) is the author/funder, who has granted bioRxiv a license to display the preprint in perpetuity. It is made available under aCC-BY 4.0 International license.

Supplementary Figures

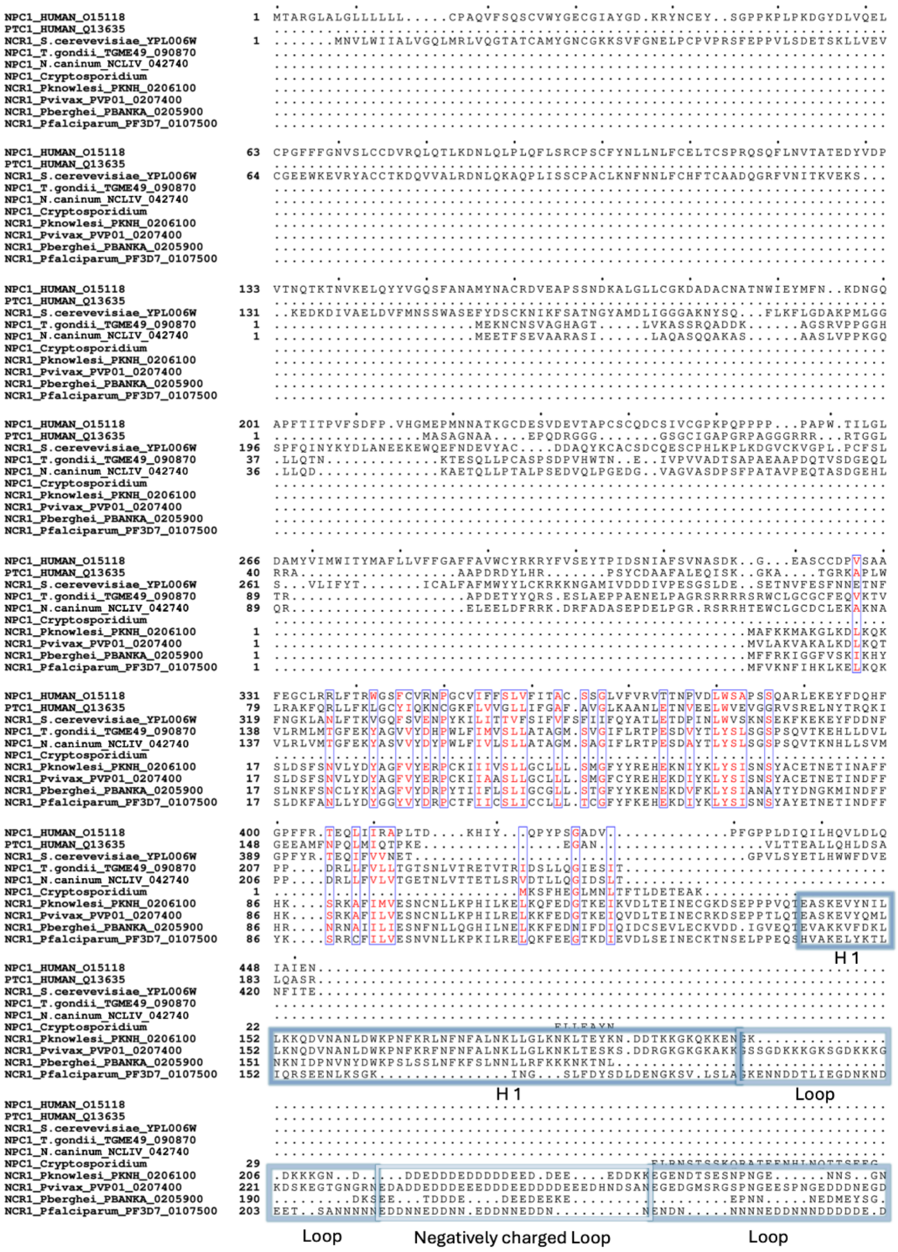

bioRxiv preprint doi: https://doi.org/10.64898/2026.03.25.714277; this version posted March 26, 2026. The copyright holder for this preprint (which was not certified by peer review) is the author/funder, who has granted bioRxiv a license to display the preprint in perpetuity. It is made available under aCC-BY 4.0 International license.

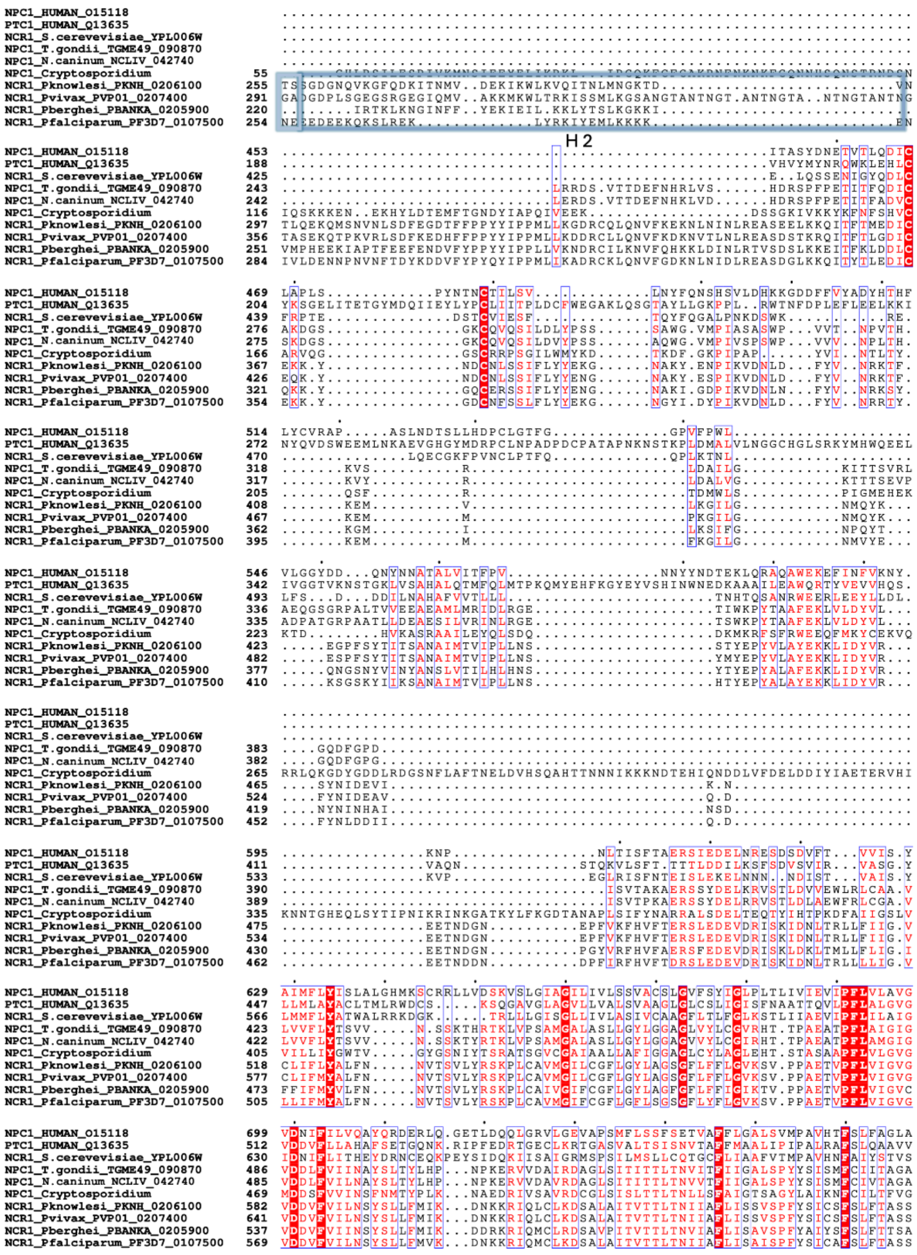

bioRxiv preprint doi: https://doi.org/10.64898/2026.03.25.714277; this version posted March 26, 2026. The copyright holder for this preprint (which was not certified by peer review) is the author/funder, who has granted bioRxiv a license to display the preprint in perpetuity. It is made available under aCC-BY 4.0 International license.

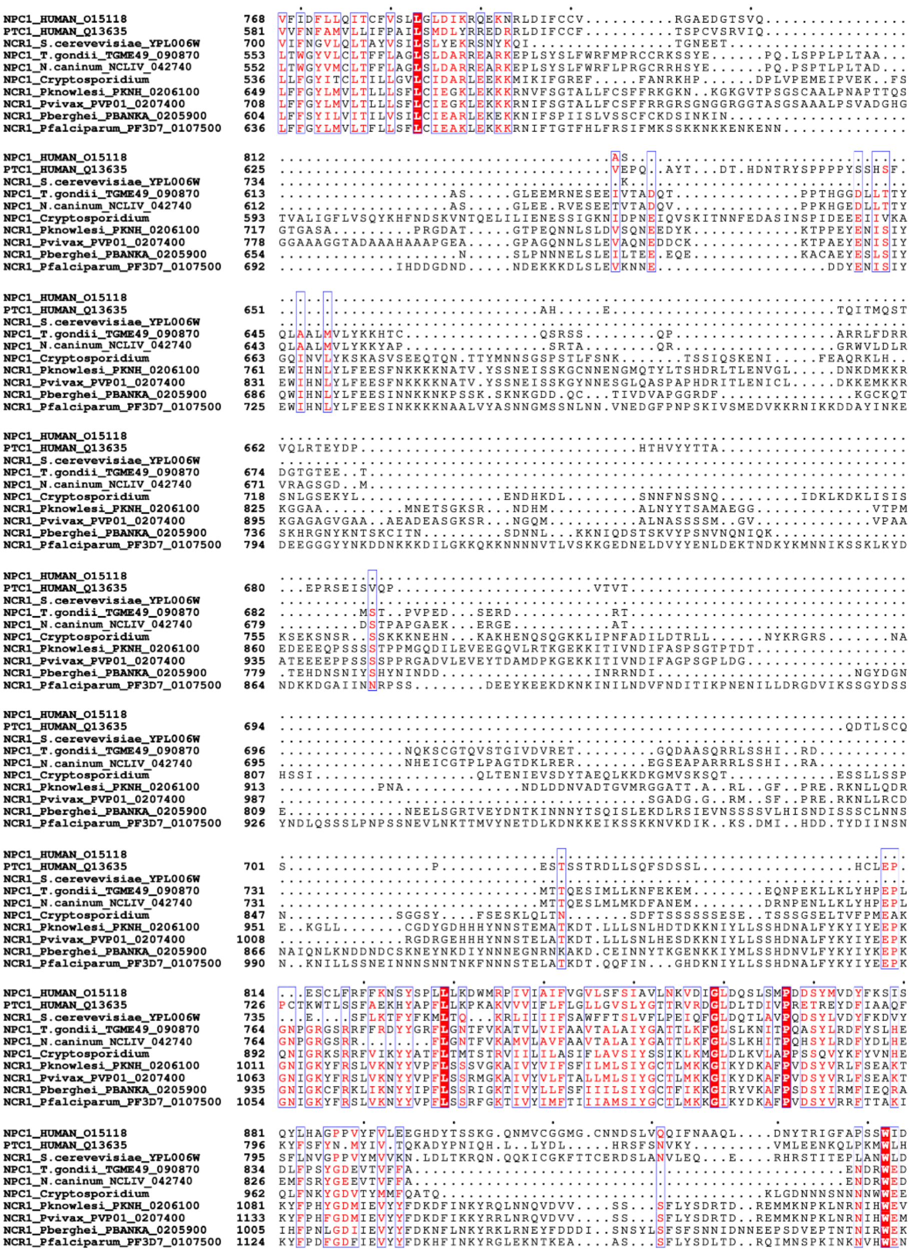

bioRxiv preprint doi: https://doi.org/10.64898/2026.03.25.714277; this version posted March 26, 2026. The copyright holder for this preprint (which was not certified by peer review) is the author/funder, who has granted bioRxiv a license to display the preprint in perpetuity. It is made available under aCC-BY 4.0 International license.

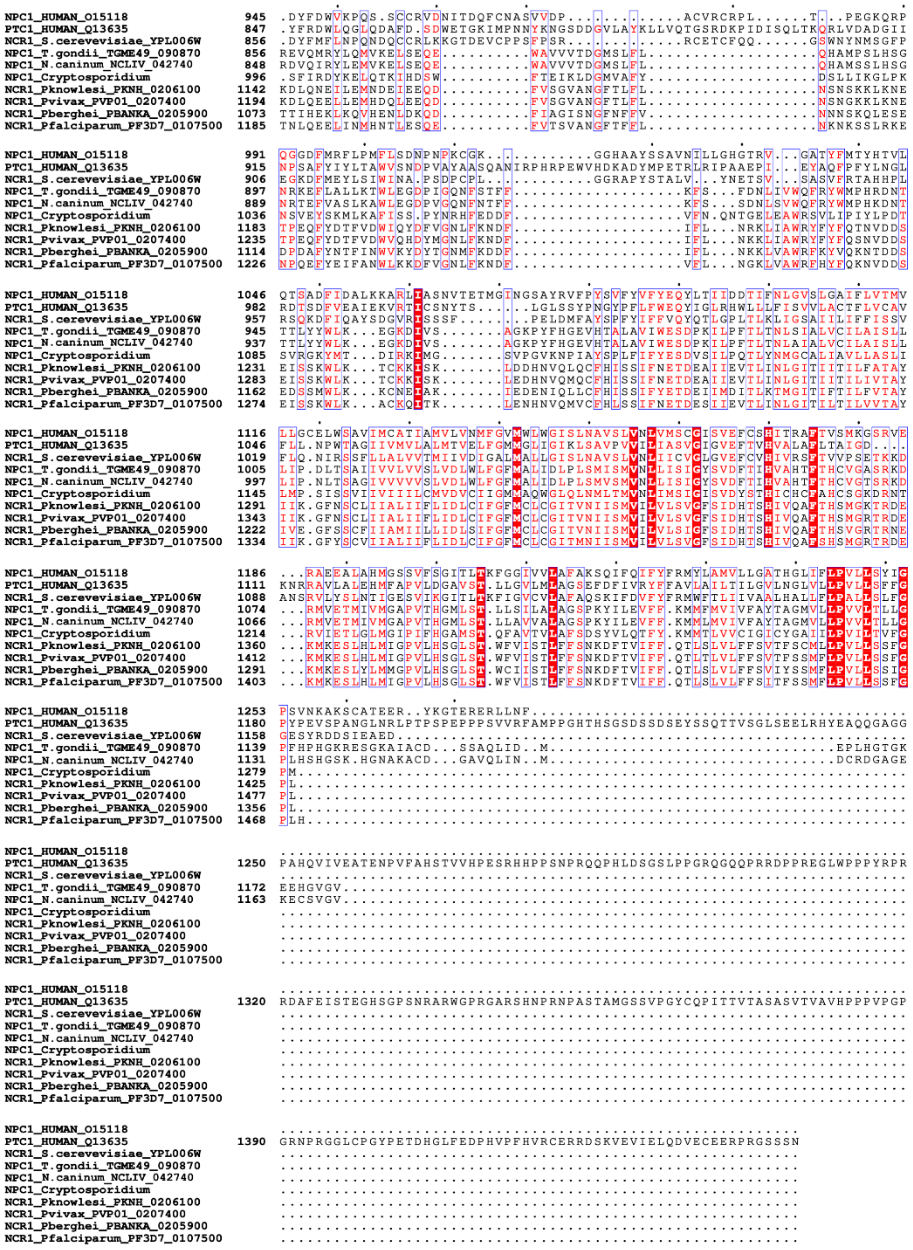

Fig. S1: The aa142-282 region in PfNCR1 consisting of amphipathic α-helices bridged by a disordered linker is unique to *Plasmodium* species. Clustal-O alignment of PfNCR1 sequence with homologs from other eukaryotes. The HLH-domain is absent in homologs from human, yeast and related parasites but is conserved across *Plasmodium* species, with presence of the negatively charged aspartic and glutamic acid repeats. Regions corresponding to Helix1, Helix2, disordered linker and negatively charged repeats in the linker are boxed in shades of blue.

bioRxiv preprint doi: https://doi.org/10.64898/2026.03.25.714277; this version posted March 26, 2026. The copyright holder for this preprint (which was not certified by peer review) is the author/funder, who has granted bioRxiv a license to display the preprint in perpetuity. It is made available under aCC-BY 4.0 International license.

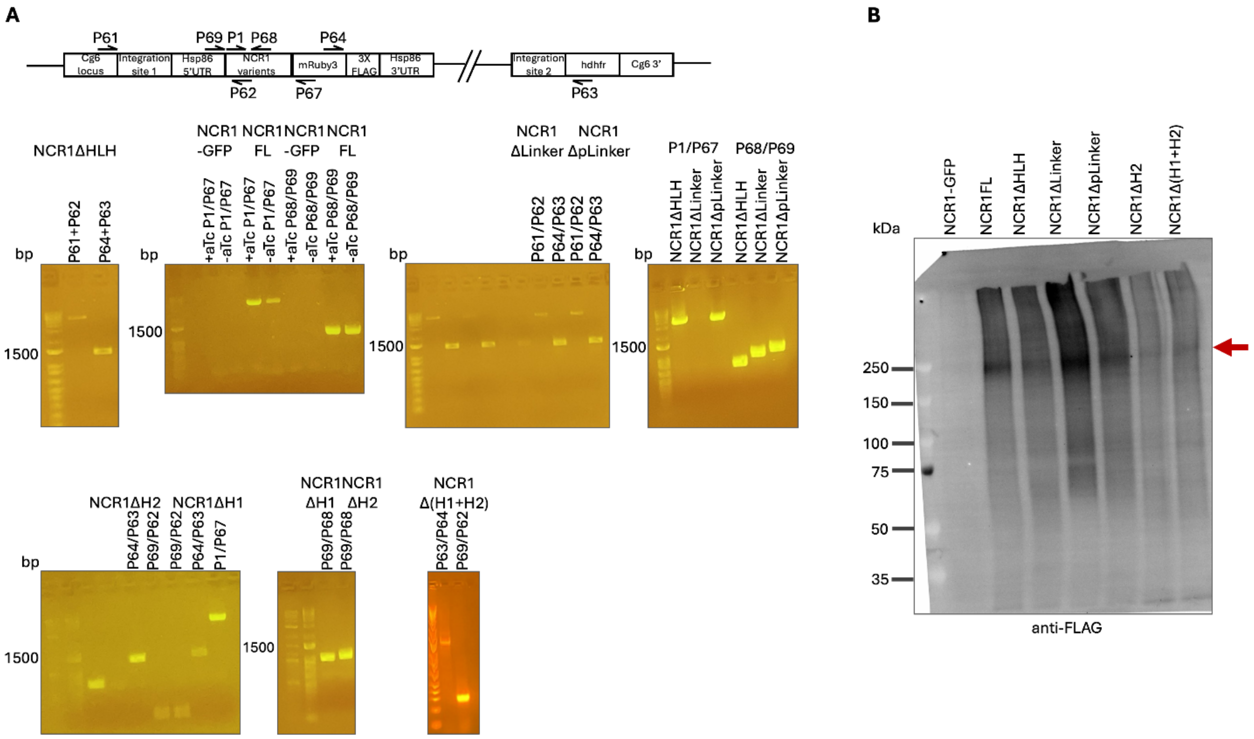

Fig. S2 Verification of expression of second copy NCR1 variants in NCR1-GFP^{apt} parasites.

A. Schematic representation of the attB × attP recombination leading to integration of ncr1 variant genes at the cg6-attB locus. PCR reactions using p61 + p62, p63 + p64, p61+p67, and p68+p69 were performed to confirm integration of the genes at the cg6-attB locus. Sequences of the primers for PCR screening are as follows: P1: 5' GAATAAACACGATTTTTTCTCGAGATGTTCGTAAAAAATTTTATACATAAATTAAAAG, P61: 5'-GAAAATATTATTACAAAGGGTGAGG , P62: 5'- GTCGTTTATGGTTTCATTCGTTTC , P63: 5'-CTCTTCTACTCTTTCGAATTC , P64: 5'- GATGATGTATCCAGCAGATG , P67: 5'-GTACGGCTGCCATACATGAACGAC, P68: 5'- CTAAATTATCAACTTTTATAGGATAATCT ATATATCCATTTC, P69: 5'- CTTCCCACATTTCGAATA AACAC.

B. Western blot analysis of the parent NCR1-GFP and second copy variants of NCR1FL, NCR1ΔHLH and HLH sub-region deletion parasite lines. Arrow indicates prominent band at ~200 kDa in the second copy variants and no band in the parent NCR1-GFP line. The expected molecular weight of NCR1FL is 200.4 kDa while that of deltaHLH, with the largest amino acid truncation, is 183.9 kDa. When compared to NCR1FL, a difference of ~16 kDa for NCR1ΔHLH and an even smaller difference in size for the sub-domain truncations remain unresolved, and they appear to co-migrate potentially due to limited resolution around ~250 kDa range.

bioRxiv preprint doi: https://doi.org/10.64898/2026.03.25.714277; this version posted March 26, 2026. The copyright holder for this preprint (which was not certified by peer review) is the author/funder, who has granted bioRxiv a license to display the preprint in perpetuity. It is made available under aCC-BY 4.0 International license.

bioRxiv preprint doi: https://doi.org/10.64898/2026.03.25.714277; this version posted March 26, 2026. The copyright holder for this preprint (which was not certified by peer review) is the author/funder, who has granted bioRxiv a license to display the preprint in perpetuity. It is made available under aCC-BY 4.0 International license.

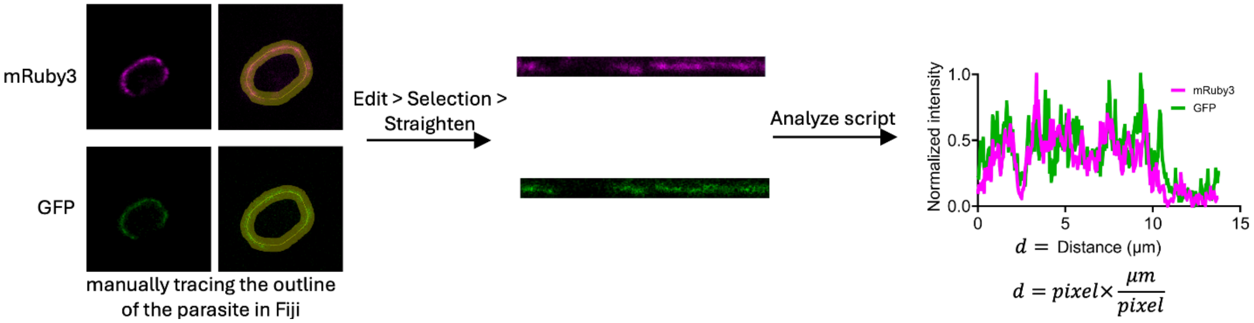

Fig. S3 Schematic of periphery unwinding for fluorescent intensity analysis.

A segmented region of interest at the parasite periphery is converted into a linearized contour of length d=pixels* (microns/pixels), enabling position-dependent plotting of GFP and mRuby3 fluorescence intensities along the membrane.

bioRxiv preprint doi: https://doi.org/10.64898/2026.03.25.714277; this version posted March 26, 2026. The copyright holder for this preprint (which was not certified by peer review) is the author/funder, who has granted bioRxiv a license to display the preprint in perpetuity. It is made available under aCC-BY 4.0 International license.

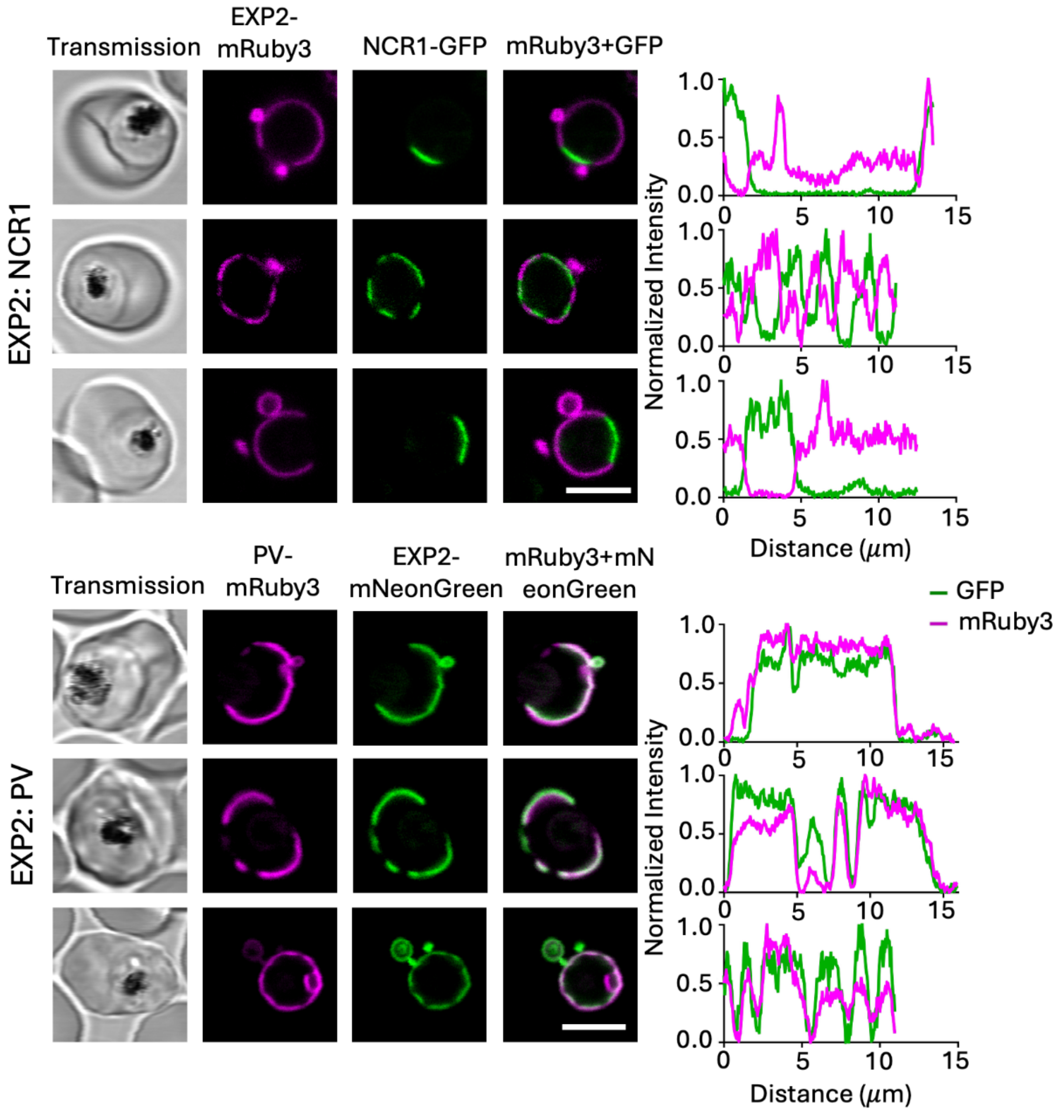

Fig. S4 Confocal images for reproduction of PCCs of previously published cells lines (Garten et al. 2020) for our current experimental setup conditions.

Confocal slice of parasites expressing NCR1-GFP::EXP2-mRuby3 (top) and EXP2-mNeongreen::PV-mRuby3 (bottom). EXP2- or PV-mRuby3 (magenta), NCR1-GFP or EXP2-mNeongreen (green). Colocalization analyzed by plotting the GFP/mNeongreen and mRuby3 fluorescence intensities measured across the parasite periphery. Scale bar: 5 µm.

bioRxiv preprint doi: https://doi.org/10.64898/2026.03.25.714277; this version posted March 26, 2026. The copyright holder for this preprint (which was not certified by peer review) is the author/funder, who has granted bioRxiv a license to display the preprint in perpetuity. It is made available under aCC-BY 4.0 International license.

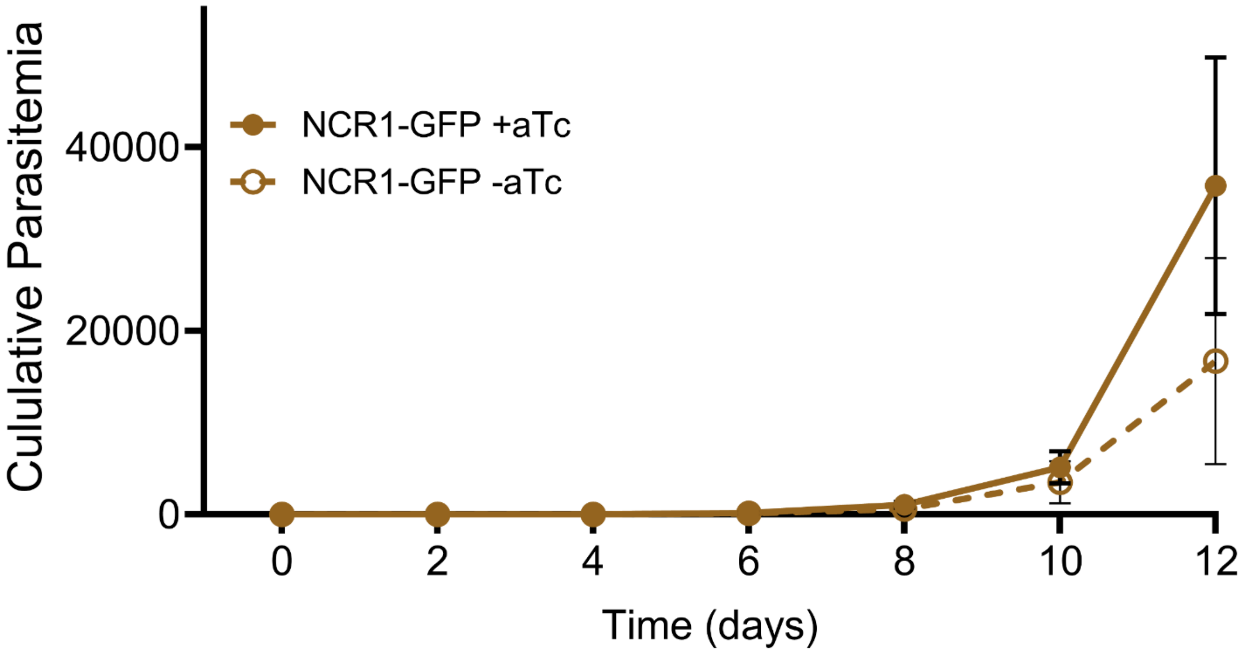

Fig. S5. Growth curve of NCR1-GFP +/-aTc.

NCR1-GFP parasites were cultured with (+ ; solid line) or without (- ; dashed line) aTc at 1% parasitemia with 2% hematocrit, sub-cultured every 48 hours. Parasite growth was monitored using a flow cytometer. The plot represents cumulative parasitemia calculated by multiplying with the dilution factor over twelve days for two biological replicates. Doubling time +aTc is 0.80 days and -aTc 0.88 days (exponential growth fit on log transformed data in GraphPad prism).

bioRxiv preprint doi: https://doi.org/10.64898/2026.03.25.714277; this version posted March 26, 2026. The copyright holder for this preprint (which was not certified by peer review) is the author/funder, who has granted bioRxiv a license to display the preprint in perpetuity. It is made available under aCC-BY 4.0 International license.

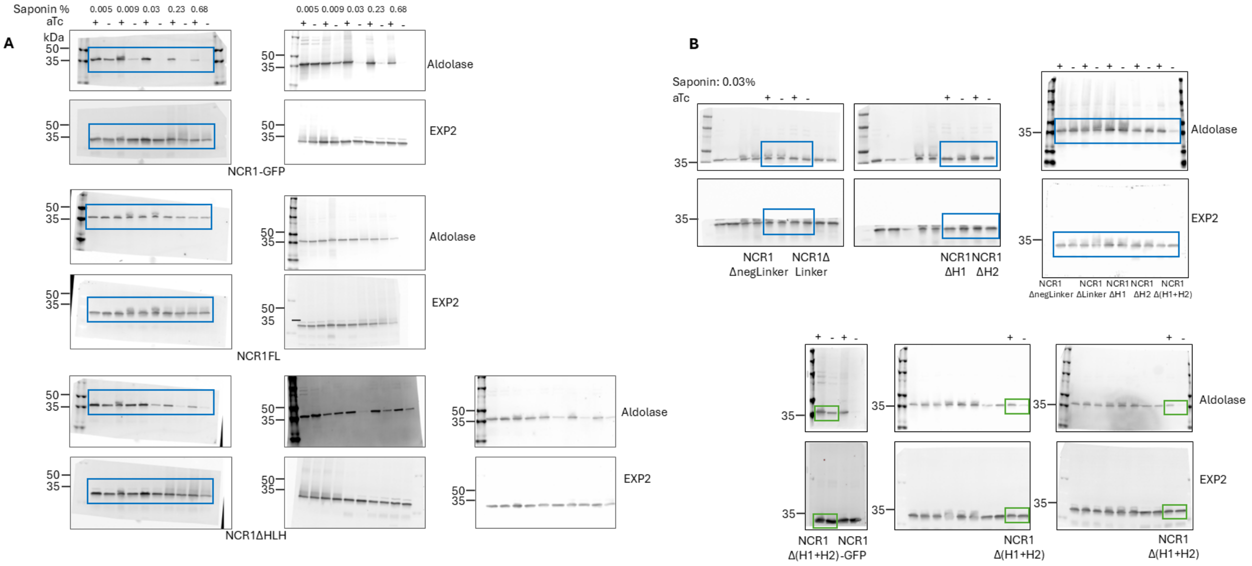

Fig. S6 Uncropped western blot images of Figs. 3A and 3C.

The blue boxes represent images included in Fig. 3 B and D. The green boxes represent biological and technical replicates for NCR1Δ(H1+H2). The other western blots are biological replicates of individual experiments.

bioRxiv preprint doi: https://doi.org/10.64898/2026.03.25.714277; this version posted March 26, 2026. The copyright holder for this preprint (which was not certified by peer review) is the author/funder, who has granted bioRxiv a license to display the preprint in perpetuity. It is made available under aCC-BY 4.0 International license.

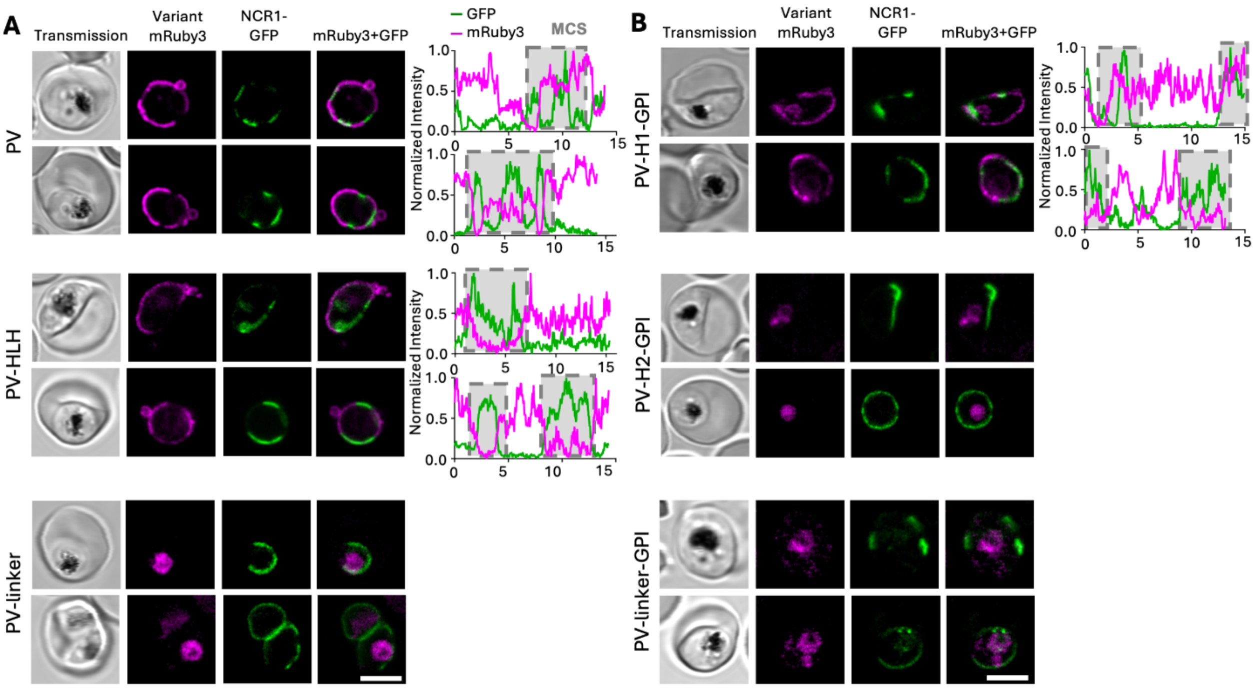

Fig. S7. Additional examples of cells from figure 4.

A. Confocal microscopy images of parasites expressing NCR1-GFP and PV targeted mRuby3 (PV-mRuby3), HLH-domain-mRuby3 (PV-HLH-mRuby3), and the disordered linker-mRuby3 (PV-Linker-mRuby3).

B. Addition of a GPI-anchor to the individual helices H1 and H2: PV-H1-mRuby3-GPI and PV-H2-mRuby3-GPI, and to the linker: PV-linker-mRuby3-GPI.

A-B: PV - relatively high: -0.26, low: -0.78; PV-HLH - average: -0.44, low: -0.79; PV-H1-GPI - relatively high: 0.21, low: -0.63. Protein domain diagrams on top of sub-panels introduce PV signal sequence tagged proteins with or without GPI expressed as second copy in the NCR1-GFP parent line. Scale bar: 5 µm.
